# Beneficial Bacteria in the Gut Microbiota May Lead to Improved Metabolic and Immunological Status in Chronic Obstructive Pulmonary Disease

**DOI:** 10.3390/medsci12030041

**Published:** 2024-08-16

**Authors:** Fabine Correia Passos, Lucas Matheus Gonçalves de Oliveira, Fabíola Ramos Jesus, Dalila Lucíola Zanette, Odilon Lobão Leal Neto, Margarida Célia Lima Costa Neves, Antônio Carlos Moreira Lemos, Gyselle Chrystina Baccan

**Affiliations:** 1 Departamento de Bioquímica e Biofísica, Instituto de Ciências da Saúde, Universidade Federal da Bahia, Salvador 40170-110, Bahia, Brazil; fabine.passos@ufba.br (F.C.P.); lucasmgo@ufba.br (L.M.G.d.O.); netinho.leall@hotmail.com (O.L.L.N.); 2 Maternidade Climério de Oliveira (MCO/EBSERH), Universidade Federal da Bahia, Salvador 40055-150, Bahia, Brazil; fabiola.jesus@ebserh.gov.br; 3 Instituto Carlos Chagas, Fundação Oswaldo Cruz, Curitiba 81350-010, Paraná, Brazil; dalila.zanette@fiocruz.br; 4 Unidade do Sistema Respiratório, Ambulatório Professor Francisco Magalhães Neto, Hospital Universitário Professor Edgard Santos, Universidade Federal da Bahia, Salvador 40110-200, Bahia, Brazil; mcneves@ufba.br (M.C.L.C.N.); acmlemos1@gmail.com (A.C.M.L.)

**Keywords:** chronic obstructive pulmonary disease, gut microbiota, cytokines, clinical markers

## Abstract

The progression of chronic obstructive pulmonary disease (COPD) is characterized by functional changes in the airways. The lung–gut axis and gut microbiota (GM) have been linked to the pathophysiology of airway diseases. Regarding COPD, studies have shown that GM alterations could be related the stages of this disease. However, the relationship between GM and clinical, biochemical and immunological parameters in patients with COPD are not well understood. The aim of this study was to compare the relative abundance of specific groups of beneficial gut bacteria between COPD patients and healthy controls (CTLs) in order to evaluate relationships with metabolic and inflammatory markers in COPD. Methods: We included 16 stable COPD patients and 16 healthy volunteer CTLs. The relative abundances of *Bifidobacterium* spp. (Bf) and *Akkermansia muciniphila* (Akk) bacteria and the Bacteroidetes and Firmicutes phyla were assessed by qPCR. Pulmonary function was evaluated by spirometry, biochemical parameters by colorimetric methods and plasma cytokine levels by cytometric bead array analysis. Results: The Firmicutes/Bacteroides ratio was related to emergency hospital visits and six-minute walk test (6MWT) results. Furthermore, the relative abundance of Bf was associated with plasma concentrations of glucose, triglycerides, HDL-C and IL-10. In addition, Firmicutes levels and the Firmicutes/Bacteroidetes ratio were associated with the IL-12/IL-10 ratio, while Akk abundance was linked to IL-12 levels. Conclusions: The present findings suggest that the abundance of beneficial bacteria in the GM could influence clinical presentation and immunoregulation in COPD.

## 1. Introduction

The gastrointestinal tract is inhabited by a wide variety of microorganisms, including bacteria, archaea, fungi and viruses [1]. The gut microbiota (GM) is mainly composed of bacteria of the Firmicutes (Fir) and Bacteroidetes (Bact) phyla, as well as Actinobacteria, Proteobacteria and Verrucomicrobia to a lesser extent [2,3]. GM bacteria are capable of producing a variety of compounds that exert important physiological effects, including the regulation of metabolism and immune responses [4,5]. The Fir phylum is composed of bacteria involved in the protection of intestinal mucosa [6]. Bact participates in metabolic conversions related to the degradation of proteins or complex sugar polymers [7] and the production of short-chain fatty acids (SCFAs) [8]. The Fir/Bact ratio is today considered an important marker of GM alterations, as previous studies identified imbalances in the Fir/Bact ratio under different clinical conditions. The main members of the Actinobacteria phylum, *Bifidobacterium* spp. (Bf), in addition to being crucial to the development of the immune system, also play fundamental roles in the development and maintenance of intestinal homeostasis, the stabilization of the intestinal barrier, and the production of SCFA [9]. *Akkermansia muciniphila* (Akk), a member of the Verrucomicrobia phylum, has been associated with a healthier metabolic state and promotes homeostasis of carbohydrate and lipid metabolism [10], as well as the generation of metabolites such as SCFA [11]. Studies have evidenced that the GM plays a key role in inflammatory response by regulating the immune system through microbial components that produce stimulatory or regulatory effector responses important for host defense, as well as microbial metabolic products that interact with host cells and influence T cells [12].

The crosstalk between the gut and the lung and its consequent impact on the pathophysiology of airway diseases has recently become the focus of intense study [13,14,15]. Evidence has shown that patients with respiratory infections or lung diseases, such as asthma, cystic fibrosis and chronic obstructive pulmonary disease (COPD), may present alterations in GM composition [16,17,18,19,20,21,22,23]. Changes in microbial diversity, mainly alterations in the abundance of beneficial gut bacteria, such as Bf, have been detected in individuals with asthma [24]. In addition, alterations in the levels of Fir and Bact have been documented in patients with cystic fibrosis and COPD [23,25,26,27]. Previous study has suggested that the GM of COPD patients is distinct from that of healthy individuals [22]. Other findings further indicate that GM composition could influence lung function parameters [16,17,23,27] during COPD progression [23]. While alterations in GM composition have been reported in COPD, relatively little is known about the implications of these alterations on metabolism and immune response. COPD is a pulmonary disease mainly caused by persistent exposure to noxious particles and gases, principally tobacco smoke, which results in alterations in lung structure and function, provoking respiratory symptoms and airflow limitations [28]. The severity of airflow limitation in COPD has been associated with the migration of neutrophils, macrophages and lymphocytes to the lung tissue [29]. Increased levels of inflammatory cytokines IL-1β, IL-6, TNF-α, IL-8 and IFN-γ are found in patients with COPD when compared to healthy individuals [30,31,32]. Furthermore, alterations in glucose and in lipid metabolism have been associated with clinical outcomes and increased risk of metabolic syndrome in these patients [33].

A systematic review previously conducted by our group identified several types of gut bacteria as being implicated in the pathophysiological mechanisms of airway diseases, including Fir and Bact phyla, Bf and Akk [15]. However, the relationship between GM composition and the pathophysiology of COPD remains unclear. The present study sought to first evaluate the relative abundance of Fir and Bact phyla, Bf and Akk, in COPD patients compared to controls (CTLs) and then determine relationships between GM makeup and clinical, metabolic and inflammatory markers in the COPD patients. To the best of our knowledge, no previous studies have attempted to specifically investigate relationships between these bacteria groups and COPD markers in COPD patients with no history of occupational exposure to toxic substances, no history of anti-inflammatory medication, immunosuppressant or corticosteroid use and without any exacerbations of lung disease in the two months prior to investigation. We identified important associations between GM composition and clinical, biochemical and immunological markers, suggesting that the GM may play a role in the pathophysiology of COPD patients, which could provide valuable insight into the development of novel study designs to more comprehensively investigate the role played by the GM in the context of COPD.

## 2. Materials and Methods

### 2.1. Study Population

The present study included 16 patients diagnosed with COPD who were recruited at the Respiratory System Service of the Professor Edgar Santos University Hospital Complex—Federal University of Bahia (HUPES-UFBA). Study controls (CTLs, n = 16) consisted of age- (±2 years) and sex-matched volunteers without a diagnosis of COPD, recruited from a public school that offers adult educational services. The exclusion criteria for both groups (COPD and CTL) were as follows: subjects with an occupational history of exposure to toxic substances, current use of antibiotics or anti-inflammatory drugs, immunosuppressive drugs, corticosteroids or hormone replacement therapy, any orthopedic, neurological or cognitive impairment, a history of digestive pathology, pulmonary disease exacerbation within the past two months or oxygen dependence. All included participants were aged between 50 and 70 years.

This study was conducted in accordance with the guidelines specified in the Declaration of Helsinki, and all procedures involving human subjects/patients were approved by the HUPES Institutional Review Board (protocol no. 4.045.818/2019). Patient participation was strictly voluntary, and all included individuals provided a signed term of consent and received guidance regarding the ethical aspects of the research performed.

### 2.2. Evaluation of Clinical Parameters

A clinical diagnosis of COPD was achieved using the Global Initiative for Chronic Obstructive Lung Disease (GOLD) criteria, considering the patient’s clinical history (dyspnea, chronic cough, expectoration, pulmonary exposure to noxious particles or gases) and spirometry test results (post-bronchodilator FEV_1_/FVC < 69% of the predicted value) [34]. Pneumotachography and Koko PFT software for Windows, version 4.1 (PDS Instrumentation, Inc., Louisville, CO, USA) were used to evaluate lung function both pre- and post-bronchodilator, and lung volumes were obtained according to the standard recommendations of the American Thoracic Society/European Respiratory Society (ATS/ERS) [35]. The six-minute walk test (6MWT) was performed according to the American Thoracic Society guidelines (Laboratories, 2002). The algorithm proposed by Iwama et al. (2009) [36] was used to predict distance on the 6MWT. Functional dyspnea was scored using the modified Medical Research Council (mMRC) scale [37]. Prognostic evaluations were performed using the body-mass index, airflow obstruction, dyspnea and exercise (BODE) Index, a multidimensional 10-point scale used to assess risk of death [38].

### 2.3. Biochemical Analyses and Cytokine Assays

Peripheral blood was collected from all COPD patients, and serum samples were stored at −20 °C. Fasting glycemia and lipoprotein levels were quantified in serum. Fasting glycemia, triglycerides, total cholesterol and HDL cholesterol (HDL-C) were measured by colorimetry using commercial kits and the Konelab 600i chemistry analyzer (Wiener Lab, Rosario, Argentina), considering the following detection limits: 0.54 mg/dL, 0.9 mg/dL, 0.63 mg/dL, 4 mg/dL, respectively. Low-density lipoprotein-cholesterol (LDL-C) was determined using the Friedewald equation (LDL-C = total cholesterol − HDL-C − Triglycerides/5).

Cytokines IL-6, IL-8, IL-10, IL-12 and TNF were quantified by a cytometric bead array using a commercial kit (BD Pharmingen, San Diego, CA, USA) using the following respective detection limits: 2.5, 3.6, 3.3, 1.9 and 3.7. Sample processing and data analysis were performed in accordance with the manufacturer’s instructions.

### 2.4. Gut Microbiota Analysis

Fecal samples were obtained from all study participants, collected in sterile plastic tubes by participants at their homes in the early morning, who then brought them to the Pneumology Service, which transported samples on ice to a laboratory for processing. DNA was extracted from each sample (200 mg, wet weight) using the QIAamp DNA StoolMini Kit (Qiagen, Hilden, Germany) following the manufacturer’s instructions. DNA concentrations were determined by absorbance at 260 nm (A260), and purity was estimated by determining the A260 to A280 ratio on a Nanodrop spectrophotometer (Nanodrop Technologies, Wilmington, DE, USA). Purified DNA was stored at −20 °C until use.

Quantitative real-time PCR (qPCR) was performed to determine the relative abundance of Fir, Bact, Bf and Akk using group-specific 16S rRNA gene primers for each bacteria species (Isogen Life Sciences, De Meern, The Netherlands) (Table 1) [39,40,41,42,43]. PCR amplification and detection were performed using a 7500 Fast Real-Time PCR System (Applied Biosystems, Foster, CA, USA). Each reaction was performed in duplicate at a final volume of 10 μL using 96-well optical-grade PCR plates sealed with optical sealing tape (Bio-Rad Laboratories, Hercules, CA, USA). Each reaction mixture (10 μL) was composed of 5 μL of SYBR Green PCR Master Mix (Applied Biosystems, Carlsbad, California, USA), 0.4 μL of each of the specific primers and 2 μL of template DNA (2 ng/μL). The thermal cycling conditions used were as follows: a single cycle at 95 °C (3 min), 30 denaturation cycles at 95 °C (15 s), primer annealing at 60 °C (30 s) and a final cycle at 95 °C (15 s). Fluorescence products were detected in the last step of each cycle. Reactions were optimized to eliminate nonspecific products or primer dimers, in accordance with melting curve analysis. A standard curve was generated for each set of primers, with the efficiency of each reaction being determined. Relative quantification was calculated by the 2^−ΔΔ^Ct method [44] using conserved 16S rRNA-specific primers (total bacteria) as the reference gene. Results are expressed as fold changes in the target gene compared to the reference gene.

### 2.5. Statistical Analyses

All statistical tests were performed using GraphPad software version 5.0 (GraphPad Software Inc., San Diego, CA, USA). Continuous variables were expressed as medians with interquartile range. Statistical differences between groups were analyzed using the Mann–Whitney test. Correlations were determined using Spearman’s rank correlation coefficient.

## 3. Results

### 3.1. Clinical, Biochemical and Immunological Markers in COPD Patients

A total of 32 subjects were included: 16 patients with COPD, and 16 sex- and age-matched healthy individuals in the control group (CTL). The median age in the COPD group was 62.50 (58.50–66.75) years, compared to 63.00 (59.50–67.00) years in the control group.

Clinical, biochemical and immunological parameters were characterized in the patients with COPD and CTLs. Table 2 lists data on pulmonary function and other disease parameters, while Table 3 details serum levels of fasting blood glucose, plasma lipoproteins and cytokine levels. Individuals with COPD were found to present lower FEV1 (%predicted), FVC (%predicted) and FEV1/FVC % than CTLs.

### 3.2. Gut Microbiota Analysis and Associations with COPD Parameters

The relative abundance of each of the bacteria species analyzed was similar between the COPD patients and controls (Table 4).

Associations between bacteria abundance in the GM and clinical, biochemical and immunological markers were investigated in the COPD patients. Regarding clinical parameters, a positive correlation was identified between hospital emergency visits and the Fir/Bact ratio (i.e., patients who presented higher numbers of visits to the emergency unit had a higher Fir/Bact ratio and lower relative levels of Bact) (Table 5). The Fir/Bact ratio was also found to be negatively associated with 6MWT, as COPD patients with higher ratios presented lower scores (Table 5). No significant associations were observed between bacteria abundance and lung functional markers or other COPD prognostic parameters (Table 5).

Bf levels were associated with several biochemical parameters (Table 6) in COPD patients, as a higher relative abundance of Bf was correlated with lower serum glucose and triglycerides levels, as well as increased levels of serum HDL-C.

Bf was also positively associated with IL-10 (Table 6), while IL-12 levels correlated positively with Akk, and the IL-12/IL-10 ratio was found to be positively correlated with Fir levels and the Fir/Bact ratio (Table 6).

## 4. Discussion

Crosstalk between the lungs and the gut has been well documented, highlighting the participation of GM in the pathophysiology of pulmonary disorders [13,14], and the influence of these alterations on clinical aspects have been evidenced in several respiratory diseases, including COPD [15,23,27]. However, the impact of GM composition in patients with COPD is not fully understood. Although previous studies have established correlations between the different stages of COPD and GM [21,27], few studies have been centered on stable COPD patients [16]. Herein, we compared the relative abundance of specific gut bacteria between healthy CTLs and COPD patients with no history of occupational exposure to toxic substances, no history of anti-inflammatory medication, immunosuppressant or corticosteroid use and without any exacerbations of lung disease in the two months prior to investigation. Our aim was to investigate associations between these bacterial groups and clinical, biochemical and immunological parameters in COPD. Our results suggest that the GM may impact metabolic and immunological parameters more than pulmonary function markers in COPD patients. However, the two main Fir and Bact phyla, as well as the Fir/Bact ratio and specific bacteria such as Bif and Akk, represent suitable targets for investigation since these have been considered as markers of dysbiosis in several others pathological conditions. In addition, members of the genus Bif and Akk are already being used in commercial applications as probiotics and may consequently emerge as promising targets for future investigations in COPD therapeutic strategies.

COPD patients present impaired lung function, as evidenced by lower values on spirometry and the six-minute walk test and higher values on the dyspnea scale and BODE index [29,45]. In severe COPD, an increased number of visits to hospital emergency centers and higher rates of hospitalization have been documented [46]. Studies previously suggested that frequency of exacerbations and hospitalizations can be related to disease severity and to a higher number of comorbidities [46]. Beyond these classical clinical markers of COPD, other parameters, such as biochemical and immunological markers, have been linked to comorbidities and/or worse disease prognosis [32,47,48,49,50,51]. In our study, COPD patients showed worse levels of biochemical markers when compared to CTLs. Some studies have demonstrated alterations in glucose and lipid metabolism in COPD patients [50,52,53,54]. Regarding cytokines, increased TNF-α, IL-1β, IL-6 and IL-8 and decreased IL-10 levels have been related to a worsening of clinical markers and increased COPD severity [51,55,56]. In sum, chronic inflammation seems to be a central mechanism underlying both respiratory symptomatology as well as COPD-associated comorbidities.

Our results revealed no differences between COPD patients and CTL individuals with respect to the relative abundance of Fir, Bact, Bf and Akk. However, some recent studies have identified distinct fecal microbiomes and metabolomes in COPD patients compared to healthy individuals [21,22]. Chiu et al. [27] investigated the relationship between GM and COPD severity using high-throughput 16S rRNA sequencing in patient stool samples, finding that while disease severity was linked to some bacteria groups, GM richness remained unaffected. In a different study involving a one-year follow-up of stable COPD patients, these same authors identified reduced GM community richness in a group of patients with worse lung function [23]. Moreover, the alpha diversity of GM, as well as Akk abundance, were found to be decreased in patients with smoking-related COPD [57]. Another study evaluated the effects of fecal microbiota inoculation from COPD patients and healthy controls into recipient mice, with subsequent exposure to smoke from biomass fuel designed to induce COPD-like changes [58]. The authors concluded that the results from their experimental model indicate that the alterations in GM similarly seen in COPD patients provoked accelerated disease progression. Other studies have reported that COPD patients face a higher risk of developing inflammatory bowel disease and increased intestinal permeability during acute exacerbations, raising the possibility that these patients may present dysbiosis [59,60,61]. Taken together, these data suggest that the GM changes evidenced in COPD may play an important role in disease progression.

Although the present study found no differences between the COPD and CTL groups regarding the relative abundance of bacteria groups in the GM, and no associations between pulmonary function parameters and GM composition were observed, important associations were seen between GM composition and the clinical, biochemical and immunological markers evaluated. The Fir/Bact ratio appeared to be associated with two disease markers (6MWT and number of emergency hospital visits). Imbalance in the Fir/Bact ratio has been associated with several human metabolic diseases, such as obesity [62], diabetes [63] and inflammatory bowel disease [64]. Other studies evaluating unhealthy populations compared to healthy adults have reported higher levels of Fir and lower levels of Bact [62]. Lee et al. (2018) [65] reported that smokers present higher proportions of Bact as well as lower proportions of Fir and Proteobacteria compared to never-smokers. However, recent research has shown that patients with grade 2–4 COPD presented a lower relative abundance of Bact compared with grade 1 COPD (21).

The present study evaluated the GM of COPD patients in a stable state (i.e., without any history of exacerbations or antibiotic use for any reason for at least two months). We thusly hypothesize that the clinical picture and systemic manifestations in these individuals may not have been sufficient to provoke changes in the relative abundance of Fir and Bact in the COPD individuals evaluated. As both systemic inflammation and oxidative stress increase during COPD exacerbations [66], we believe that these COPD-associated factors may contribute to changes in GM in patients with differing degrees of disease severity, but perhaps not in stable patients.

Regarding biochemical markers, our results revealed negative associations between the relative abundance of Bf and glucose as well as triglycerides, yet a positive association was observed between Bf and HDL-C serum levels, indicating that this bacterial group may be associated with metabolic profile alterations in the COPD patients studied herein. Diverse species present in the GM represent important modulators of glucose [67] and lipid metabolism [68]. Previous studies have shown that patients with type 2 diabetes present lower Bf levels compared to healthy individuals [69]. In addition, the relative abundance of Bf and Akk species was found to correlate positively with HDL-C and negatively with fasting glucose levels in lean women, yet these species did not correlate with triglycerides [70]. While the precise mechanism by which Bf influences lipid and glucose homeostasis is not yet fully understood, several studies suggest that it may participate in reducing low-grade endotoxemia, increasing short-chain fatty acid levels and provoking alterations in bile acid metabolism [71]. Data from experimental models indicate that the action of this probiotic on metabolism may be mediated by the stimulation of short-chain fatty acids and glucagon-like peptide-1 (GLP-1) production, as well as improved GM balance [72,73,74]. Probiotic intervention studies have reinforced the notion of beneficial Bf-induced effects on carbohydrate and lipid metabolism [75,76]. The use of probiotics containing diverse Bf species has resulted in significant improvements on total cholesterol, triglyceride and fasting blood glucose levels [75,77]. While it remains unclear exactly how Bf levels in the GM ultimately affect COPD patients, it is possible that achieving a relatively balanced abundance of Bf could contribute to better metabolic control in these patients, which could in turn favorably impact the progression of comorbidities.

Many components of the GM are considered important modulators of immune response, both local and systemic, responsible for driving the differentiation of naïve T cells and modulating cytokine production [12,78,79]. The results of this study indicate that COPD patients with higher levels of Fir or an increased Fir/Bact ratio exhibited a higher IL-12/IL-10 ratio, which could be indicative of increased inflammation. The importance of changes in the abundance of Fir and Bact phyla and the associated ratio has been evidenced in several studies on obesity [39,80]. Obese individuals present an increased Fir/Bact ratio, which seems to be related to higher serum levels of lipopolysaccharide (LPS) and inflammatory cytokines [80,81]. Studies involving obese individuals or employing experimental obesity models have reported an association between higher Fir/Bact ratios in obesity and the development of chronic low-grade inflammation [80,81]. In vitro stimulation of human peripheral blood mononuclear cells using Gram-positive bacteria induced nine times greater IL-12 production compared to stimulation with Gram-negative bacteria, which, in turn, stimulated three times higher IL-10 production than Gram-positive bacteria [82]. This finding could explain the associations observed herein between both Fir and Fir/Bact in comparison to IL-12/IL-10 levels, since most bacteria are Gram-positive, which could imply enhanced inflammatory potential in COPD. As inflammation has been linked to worse clinical parameters in COPD, a more inflammatory environment could help explain the observed associations detected between the Fir/Bact ratio and number of hospital emergency visits described in our patients. IL-12 is a cytokine that is involved in the induction of T-helper cell type 1 immune responses and also modulates the inflammatory response [83]. IL-12 is frequently increased in patients with COPD and upregulated in response to cigarette smoke exposure [84,85]. Surprisingly, we identified a positive correlation between Akk and IL-12 levels. Akk species are involved in host immunological homeostasis within the gut mucosa and the improvement of gut barrier function [11]. A study comparing GM composition among smokers and nonsmokers with Crohn’s disease found a reduced relative abundance of Akk in smokers [86]. It has been shown that in vitro stimulation of human peripheral blood mononuclear cells with Akk induced higher IL-10 production [11]. An experimental model of COPD involving mice exposed to cigarette smoke revealed that an oral gavage of Akk resulted in decreased lung tissue injury and inflammatory infiltration, with lower levels of IL-17, IL-6 and TNF-a in peripheral blood as well as improvements in autophagic markers [57].

The present study identified a positive association between the relative abundance of Bf and IL-10 levels, which could be relevant considering the importance of this regulatory cytokine in COPD [56,87]. This association could also serve to explain the relationship between Bf and the better metabolic profile observed in our COPD patients. Human intestinal mucosa-associated Bf species were shown to considerably downregulate pro-inflammatory cytokines IL-6 and IL-12 and upregulate the regulatory cytokine IL-10 in a monocytic cell line [88]. Other studies have documented the effects of different Bf strains on the in vitro production of IL-10 by peripheral blood mononuclear cells [89,90]. Verma et al. [91] identified that components from the cell wall of *B. bifidum* could be responsible for inducing Foxp3 + T_reg_cells in the intestine. In an experimental study, the oral administration of *B. breve* was shown to increase the number of IL-10-producing Foxp3^−^CD4 ^+^ T cells in the large intestine [92]. Similarly, in an experimental model of chronic allergic asthma, *B. breve* induced regulatory T cells in lung tissue, as evidenced by increased IL-10 and Foxp3 transcription [93]. Another experimental model of COPD indicated that the use of a probiotic containing Bf strains resulted in reduced macrophage migration, less severe airway remodeling and lower inflammatory cytokine production [94,95]. These aspects indicate that the beneficial effects induced by Bf in COPD patients may occur due to the upregulation of IL-10.

Although high-throughput sequencing and metagenomic analysis are important, as these approaches provide information about the entire microbiome, the obtained results are often difficult to understand, much less to apply in clinical practice. Here, we employed a relatively simple technique capable of demonstrating that the analysis of relatively few GM groups can provide an indication of dysbiosis, as well as inform inflammatory status and possibly aid in selecting the therapeutic use of probiotics to treat COPD. Our results are in line with the data obtained by other authors, serving to indicate that the Bact phylum may be related to improved lung function and more favorable COPD evolution. Meanwhile, the Fir phylum was associated with an opposite [23,27] situation, with worse lung function and evolution. One of the reasons for this finding could be the association of Fir with a higher inflammatory status (IL-12/IL-10).

The results reported herein should be considered in the light of some limitations. Our sample size was limited due to restrictive study criteria, as patients were only included at the time of diagnosis and none were using antibiotics or corticosteroids, since the use of antibiotics or other drugs could constitute a confounding factor in GM analysis. Our study did not examinate the overall abundance of bacteria phyla, as advanced analyses of the GM in this population were unfortunately not performed due to budgetary limitations. As the intestinal microbiota is composed of complex microbial communities, its investigation involves the use of advanced methodological approaches and significant expense. Nonetheless, more accessible approaches are necessary to identify changes in the microbiota in COPD patients. Herein, the quantitative real-time PCR (qPCR) technique was performed to determine relative abundance using 16S rRNA gene-specific primers. Despite this limitation, we believe that our study will provide a better understanding of the relationship between GM and clinical, immunological and biochemical markers of COPD, offering insight that could assist in the development of designs for new studies. In addition, it is important to note that we could not guarantee that the included CTL individuals were not exposed to other factors that could negatively impact GM composition.

## 5. Conclusions

The evidence presented in the present study suggests that the GM may play a role in the pathophysiology of COPD, considering associations between the relative abundance of some GM bacteria and clinical and biochemical biomarkers of this disease, as well as cytokine levels. Our results revealed that the abundance of certain bacterial species in the GM may impact metabolic and immunological parameters more than pulmonary function markers. Higher levels of Bf and a low Fir/Bact ratio seem to be related to more favorable immunoregulation and with the improvement of the biochemical profile. Further studies will be required to more comprehensively understand the mechanisms underlying the interplay between the GM and the immune system in the context of COPD.

## Figures and Tables

**Table 1:** Primers used to quantify bacterial populations by quantitative PCR.

Bacterial Group	Oligonucleotide Sequence	Product Size (bp)	Annealing Temp (°C)	Ref.
Total bacteria (16S)	F: 5′ACTCCTACGGGAGGCAGCAG3′ R: 5′ATTACCGCGGCTGCTGG3′	200	60	[38]
Phylum Firmicutes	F: 5′ATGTGGTTTAATTCGAAGCA3′ R: 5′AGCTGACGACAACCATGCAC3′	126	60	[40,41]
Phylum Bacteroidetes	F: 5′CATGTGGTTTAATTCGATGAT3′ R: 5′AGCTGACGACAACCATGCAG3′	126	60	[40,41]
*Bifidobacterium* spp.	F: 5′GCGTGCTTAACACATGCAAGTC3′ R: 5′CACCCGTTTCCAGGAGCTATT3′	126	60	[42]
*A. muciniphila*	F: 5′CAGCACGTGAAGGTGGGGAC3′ R: 5′CCTTGCGGTTGGCTTCAGAT3′	327	60	[43]

Legend: forward (F), reverse (R).

**Table 2:** Clinical characteristics of the COPD patients (n = 16).

Clinical Markers	COPD	CTL
FEV_1_ %pred, (%); median (IQR)	46.00 (32.00–58.00)	95.50 (85.00–108.00) *
FVC %pred, (%); median (IQR)	65.00 (49.00–68.00)	81.00 (73.00–90.25) **
FEV_1_/FVC, (%); median (IQR)	65.00 (46.00–69.00)	92.75 (87.20–97.85)
6MWT (%predicted)	71.66 (61.05–82.17)	---
mMRC dyspnea scale	3.00 (2.00–3.00)	---
BODE index	4.00 (3.00–7.00)	---
Emergency visits	2.00 (0.75–5.75)	---
Hospitalization (days)	0.50 (0.00–1.25)	---

Note: Data expressed as medians (interquartile range) and compared using the Mann–Whitney U test. *p* values < 0.05 considered statistically significant. IQR, interquartile range (25th–75th percentile); FEV1: forced expiratory volume in one second (%predicted); FVC: forced vital capacity (%predicted); FEV1/FVC: ratio of forced expiratory volume in the first second to forced vital capacity; 6MWT: six-minute walk test; mMRC: modified Medical Research Council; BODE: body-mass index, airflow obstruction, dyspnea and exercise capacity.* *p*  <  0.01 compared to COPD; ** *p*  <  0.001 compared to COPD.

**Table 3:** Biochemical markers and cytokine levels in the COPD patients (n = 16).

Parameter	COPD	CTL
**Glucose (mg/dL)**	112.00 (90.25–130.5)	103.2 (88.75–119.8)
**Triglycerides (mg/dL)**	129.50 (92.30–186.00)	117.7 (85.70–160.6)
**Total cholesterol (mg/dL)**	211.50 (169.8–230.5)	192.5 (169.8–222.0)
**HDL-C (mg/dL)**	46.0 (44.00–55.00)	52.00 (43.13–65.70)
**LDL-C (mg/dL)**	128.00 (95.25–150.6)	100.70 (95.00–141.0)
**IL-6 (pg/mL)**	3.82 (3.69–8.13)	---
**IL-8 (pg/mL)**	6.05 (5.30–7.65)	---
**IL-10 (pg/mL)**	8.23 (8.23–8.49)	---
**IL-12 (pg/mL)**	9.91 (9.18–10.92)	---
**TNF (pg/mL)**	12.66 (9.61–18.02)	---
**IL-6/IL-10 ratio**	0.48 (0.45–0.98)	---
**IL-8/IL-10 ratio**	0.73 (0.64–1.01)	---
**IL-12/IL-10 ratio**	1.29 (1.17–1.34)	---
**TNF/IL-10 ratio**	1.92 (1.08–2.37)	---

Note: Data expressed as medians (interquartile range) and compared using the Mann–Whitney U test. *p* values < 0.05 considered statistically significant. HDL-C: high-density lipoprotein-cholesterol, LDL-C: low-density lipoprotein-cholesterol.

**Table 4:** Relative quantification of GM bacteria in patients with COPD (n = 16) and controls (CTLs) (n = 16).

Bacteria (Fold-Change) ^a^	COPD	CTL	*p*
**Firmicutes**	1.04 (0.49–1.81)	0.95 (0.46–1.82)	0.78
**Bacteroidetes**	0.77 (0.52–1.29)	0.93 (0.50–1.87)	0.61
**Firmicutes/Bacteroidetes ratio**	1.64 (0.39–2.92)	1.32 (0.49–1.89)	0.46
***Bifidobacterium* spp.**	1.39 (0.20–13.55)	1.59 (0.59–4.34)	0.79
***A. muciniphila***	2.18 (0.16–14.32)	1.00 (0.053–2.4)	0.30

Note: Data expressed as medians (interquartile range) and compared using the Mann–Whitney U test. *p* values < 0.05 considered statistically significant. ^a^ Relative quantification performed using the comparative 2^−∆∆Ct^ method.

**Table 5:** Correlations between relative GM bacteria abundance and clinical markers in COPD patients (n = 16).

Parameter	Phylum Firmicutes		Phylum Bacteroidetes		Firmicutes/Bacteroidetes		*Bifidobacterium* spp.		*A. muciniphila*	
	R	*p*	r	*p*	r	*p*	r	*p*	r	*p*
**FEV_1_** **%predicted**	−0.2033	0.2527	0.0373	0.4495	−0.1868	0.2706	0.0545	0.4405	−0.2545	0.2255
**FVC** **%predicted**	−0.1928	0.2594	0.0618	0.4168	−0.2121	0.2433	0.3526	0.1588	−0.0548	0.4323
**FEV_1_/FVC %**	−0.2897	0.1553	−0.1156	0.3470	−0.1950	0.2616	−0.4502	0.0958	−0.2294	0.2404
**6MWT** **%predicted**	−0.3099	0.1403	0.2571	0.1774	**−0.5879**	**0.0190**	0.0818	0.4055	−0.2273	0.2517
**mMRC**	0.2584	0.1997	0.2718	0.1736	0.1818	0.2761	−0.2774	0.2189	−0.1430	0.1768
**BODE index**	0.3971	0.0894	−0.298	0.4597	0.3942	0.0913	−0.1228	0.3677	0.2343	0.2416
**Emergency visits**	0.4983	0.0508	**−0.5631**	**0.0225**	**0.6431**	**0.0120**	0.1532	0.3469	0.1739	0.3031
**Hospitalization (days)**	0.3751	0.1137	−0.0481	0.4380	0.2970	0.1743	0.1287	0.3707	0.2585	0.2193

**Note:** r: Spearman’s correlation coefficient.

**Table 6:** Correlations between relative GM bacteria abundance and serum biochemical markers/cytokine levels in COPD patients.

Cytokines	Phylum Firmicutes		Phylum Bacteroidetes		Firmicutes/Bacteroidetes		*Bifidobacterium* spp.		*A. muciniphila*	
	R	*p*	r	*p*	r	*p*	r	*p*	r	*p*
**Glucose (mg/dL)**	−0.4549	0.0522	0.0750	0.3953	−0.3978	0.0795	**−0.6364**	**0.0176**	−0.2818	0.2012
**Triglycerides (mg/dL)**	−0.2640	0.1773	0.2539	0.1986	−0.2970	0.1512	**−0.6273**	**0.0194**	0.1185	0.3639
**Total cholesterol (mg/dL)**	0.1562	0.2955	−0.3414	0.1065	0.3432	0.1148	0.1000	0.3849	0.4601	0.0780
**HDL-C (mg/dL)**	0.0599	0.4195	−0.3783	0.0822	0.2042	0.2419	**0.7051**	**0.0077**	0.3945	0.1148
**LDL-C (mg/dL)**	0.2618	0.1814	−0.3128	0.1282	0.4092	0.0731	0.1276	0.3543	0.4510	0.0825
**HDL-C/LDL-C**	−0.1209	0.3409	0.1036	0.3567	−0.2176	0.2275	−0.1182	0.3646	−0.2545	0.2255
**IL-6 (pg/mL)**	−0.4636	0.1155	0.0678	0.4312	−0.2928	0.2408	−0.2883	0.2653	0.07207	0.4444
**IL-8 (pg/mL)**	−0.3293	0.1707	0.0411	0.4523	−0.2500	0.2430	0.1786	0.3508	0.2185	0.2856
**IL-10 (pg/mL)**	−0.1158	0.2211	−0.3360	0.1428	−0.0315	0.4633	**0.6390**	**0.0320**	0.3303	0.1971
**IL-12 (pg/mL)**	0.4469	0.0634	−0.3157	0.1358	0.4110	0.0815	0.5000	0.0706	**0.6220**	**0.0301**
**TNF (pg/mL)**	0.2371	0.2533	−0.227	0.4735	0.1277	0.3626	0.2162	0.3207	0.4048	0.1634
**IL-6/IL-10**	0.3473	0.1907	0.0920	0.4069	−0.2515	0.2740	−0.3571	0.2158	−0.1071	0.4198
**IL-8/IL-10**	−0.4268	0.1211	0.3465	0.1633	−0.4100	0.1365	−0.1429	0.3800	−0.1557	0.3461
**IL-12/IL-10**	**0.5604**	**0.0384**	−0.1786	0.2893	**0.5877**	**0.0286**	0.2510	0.2573	0.2500	0.2603
**TNF/IL-10**	−0.1429	0.3913	0.1905	0.3257	−0.2143	0.3223	0.7000	0.0941	0.3929	0.1978

**Note:** r: Spearman’s correlation coefficient.

## Data Availability

The data that support the findings of this study are available from the corresponding author upon reasonable request.
